# MEASUREMENT OF SOCIAL NETWORKS OF ELDERS USING TECHNOLOGIES IN THE CONTEXT OF HEALTH AND SOCIAL CARE: A SCOPING REVIEW

**DOI:** 10.1093/geroni/igz038.280

**Published:** 2019-11-08

**Authors:** Sijia Wei, Eleanor S McConnell, Kayla Wright-Freeman, Amanda Woodward, Bada Kang, Kirsten N Corazzini

**Affiliations:** 1 1. Duke University 2. Duke University School of Nursing JBI-Affiliated Group, Durham, North Carolina, United States; 2 Duke University and Geriatric Research, Education, and Clinical Center, Durham Veterans Affairs Health Care System, Durham, North Carolina, United States; 3 Duke University, Durham, North Carolina, United States

## Abstract

Social networks impact the health and wellbeing of older adults. The importance of social networks drives the need to reliably measure social networks. Advancements and innovations in the internet, electronic and digital devices, social media and health care technology enriches our ability to collect social network and health data to overcome limitations in social network measurement. This scoping review will review approaches utilizing technology to assist the measurement and analysis of social networks among older adults in the context of health and social care. Joanna Briggs Institute methodology was followed; PubMed (MEDLINE), Sociological Abstracts, SocINDEX, CINAHL, and Web of Science were searched for related articles. Conference abstracts and proceedings were included. We discuss the gaps and advances in measurement of social networks of older adults using technology and implications for future research in social networks of older adults as a lever for improving health and well-being.

